# Resilience and mental health problems in children and adolescents who have been victims of violence

**DOI:** 10.11606/S1518-8787.2019053000391

**Published:** 2019-01-18

**Authors:** Natália Amaral Hildebrand, Eloisa Helena Rubello Valler Celeri, André Moreno Morcillo, Maria de Lurdes Zanolli

**Affiliations:** I Universidade Estadual de Campinas. Faculdade de Ciências Médicas. Programa de Pós-Graduação em Saúde da Criança e do Adolescente. Campinas, SP, Brasil; II Universidade Estadual de Campinas. Faculdade de Ciências Médicas. Departamento de Psicologia Médica e Psiquiatria. Campinas, SP, Brasil; III Universidade Estadual de Campinas. Faculdade de Ciências Médicas. Departamento de Pediatria. Campinas, SP, Brasil

**Keywords:** Child Adolescent, Resilience, Psychological, Violence, Risk Factors, Family Relations, Mental Health, Criança, Adolescente, Resiliência Psicológica, Violência, Fatores de Risco, Relações Familiares, Saúde Mental

## Abstract

**OBJECTIVE:**

To understand the process of resilience (social support and resources of the family environment) and the chance of mental health problems in children and adolescents (9–16 years) who have been victims of domestic violence, assisted in specialized services (Group 1 – G1) and in school services without reports of domestic violence (Group 2 – G2).

**METHODS:**

Various semi-structured instruments were applied to the pairs (guardian and child or adolescent): the Strengths and Difficulties Questionnaire (SDQ); the Resiliency Scales for Children and Adolescents (RSCA), including Scale I (SI – sense of control), Scale II (SII – relationship skills) and Scale III (SIII – emotional reactivity); the Social Support Appraisals; the Home Environment Resources Scale and a questionnaire created by the authors to characterize the population.

**RESULTS:**

There was no difference in the prevalence of resilience between G1 and G2. Children and adolescents of both groups had a higher chance of low resilience in the absence of perception of social support from the teacher (SI; SIII) and other people in the community (SI; SII). Girls had higher chance of low resilience (SIII). The establishment of routine or rules in the lives of the children and adolescents facilitated the development of resilience (SIII). In G1, the prevalence of mental health problems was 65% for the self-application version of the SDQ for children and adolescents (SDQ/CA) and 54% for the version answered by the guardians (SDQ/G). In G2, it was 33% for SDQ/CA and 37.9% for SDQ/G. Domestic violence against children and adolescents was a risk factor for the development of mental disorders (SDQ/G). Subjects with low resilience (SI) had a higher chance of developing mental health problems (SDQ/CA). Despite originating from the same regions, the groups had socioeconomic differences, which showed no relationship with resilience.

**CONCLUSIONS:**

The quality and perception of social support and resources present in the home environment may have facilitated the development of resilience in the studied children and adolescents. Violence may have increased the chance of mental health problems, domestic violence being an aggravating factor. There is need for research on aspects that predict resilience and investment in intervention strategies for this population, as a way to promote mental health.

## RESUMO

**OBJETIVO:**

Compreender o processo de resiliência (suporte social e recursos do ambiente familiar) e a chance de problemas de saúde mental em crianças e adolescentes (9–16 anos) vítimas de violência doméstica acompanhados em serviços especializados (Grupo 1 – G1) e em escolares sem relatos de situações de violência doméstica (Grupo 2 – G2).

**MÉTODOS:**

Diversos instrumentos semiestruturados foram aplicados às díades (responsável e criança ou adolescente): *Strengths and Difficulties Questionnaire* (SDQ); *Resiliency Scales for Children and Adolescents* (RSCA), incluindo a Escala I (EI – sentido do controle), a Escala II (EII – capacidade de relacionamento) e a Escala III (EIII – reatividade emocional); *Social Support Appraisals*; Inventário de Recursos no Ambiente Familiar e um questionário elaborado pelos autores para caracterizar a população.

**RESULTADOS:**

Não houve diferença na prevalência de resiliência entre G1 e G2. As crianças e adolescentes de ambos os grupos tiveram maior chance de baixa resiliência na ausência de percepção do suporte social do professor (EI; EIII) e de outras pessoas da comunidade (EI; EII). Meninas apresentaram maior chance de baixa resiliência (EIII). O estabelecimento de rotina ou regras na vida das crianças e adolescentes facilitou o desenvolvimento da resiliência (EIII). No G1 a prevalência de problemas de saúde mental foi de 65% pela versão de autoaplicação do SDQ para crianças e adolescentes (SDQ/CA) e de 54% pela versão respondida pelos responsáveis (SDQ/R). No G2 foi de 33% pelo SDQ/CA e de 37,9% pelo SDQ/R. A violência doméstica infanto-juvenil foi fator de risco para o desenvolvimento de transtornos mentais (SDQ/R). Os sujeitos com baixa resiliência (EI) apresentaram maior chance de problemas de saúde mental (SDQ/CA). Embora provenientes das mesmas regiões, os grupos apresentaram diferenças socioeconômicas, as quais não apresentaram relação com a resiliência.

**CONCLUSÕES:**

A qualidade e percepção do suporte social e de recursos presentes no ambiente familiar podem ter facilitado o desenvolvimento da resiliência nas crianças e adolescentes estudados. A violência pode ter aumentado a chance de problemas de saúde mental, sendo a violência doméstica um agravante. Há necessidade de pesquisas sobre os aspectos preditores de resiliência e de investimento em estratégias de intervenção para esta população, como forma de promover a saúde mental.

## INTRODUCTION

Social violence, a global public health problem, directly impacts the health of individuals, regardless of social class. Along with accidents, violence is the third cause of mortality in the general population and the first among children and adolescents^a^. In Brazil, the rate of social and community violence is very high, and more than 30% of the population lives below the poverty threshold. However, the impact of violence and emotional, cognitive and material deprivation on people's lives is still not known in its entirety, especially regarding the psychosocial and subjective aspects^1^ ^,^ ^2^.

The data on domestic violence are also concerning: one in four children suffers physical violence and one in five girls has suffered sexual abuse^b^, Brazil being the fourth country with the highest child murder rate among the 92 countries analyzed^c^. Domestic violence against children and adolescents (DVCA) or child abuse is defined as any form of abuse, negligence, violation of rights or physical, psychological or sexual violence committed by an adult with responsibilities in relation to that growing individual^d^.

In Brazil, the limited structure of the services of assistance to victims of violence, in association with underreporting, makes it difficult to obtain safe incidence estimates. There are few complaints because family members, members of the community, professionals (in the fields of health, education and social assistance, among others) and the victims themselves, out of fear or for other reasons, turn a blind eye to the abusive dynamics. This phenomenon is called “conspiracy of silence”^1^ ^,^ ^a^ ^,^ ^e^.

The recurring assaults and abuses have serious consequences for the child or adolescent and negatively affect their physical, cognitive, emotional, and social development^2^. Studies^1^ ^-^ ^3^ indicate that experiencing situations of abuse during childhood or adolescence can be a risk factor for the development of mental health problems that, if not treated, can lead to serious consequences in adult life.

Exposure to situations of social violence at school or in the community may have a strong impact on the lives and health of children and adolescents, from physiological and psychological changes to impairment of their perspectives for the future and personal development. Some of the most commonly observed symptoms are behavioral and mood changes as well as post-traumatic stress disorder^1^.

Many subjects, however, manage to subdue these difficulties. Since 1950, longitudinal studies have revealed that some people, despite having been exposed to environments with multiple psychosocial risks, were able to stay emotionally healthy. A new field of study thus emerged, based on the concepts of resilience and vulnerability^4^ ^,^ ^5^ ^,^ ^f^. The concept of resilience has been used in various fields of knowledge. It is originally a concept of Physics, being defined as the ability of a particular body to regain its properties after having had its primary form modified by a foreign agent^6^. In the Humanities and Biological sciences, it is associated with the concept of vulnerability (which is also in process of construction): a set of collective and contextual aspects that can increase an individual's susceptibility to afflictions or diseases – broader than the concept of “risk”, which encompassed the subject's individual issues only. Therefore, the public services’ structure and the availability or lack of resources intended for the care and protection of the population becomes essential for understanding a subject's vulnerability^7^ ^,^ ^g^.

This study has as theoretical references the construct of resilience and the concept of safety or support network – or of child care services, even. The former is understood here as a phenomenon with a transitional character, mediated by individual capacities and associated with the subject's socio-cultural-history context, emphasizing the importance of sharing and redefining experiences. The latter refers to the group of people and systems with close and meaningful relationships with the subject, being either constant or dynamic^8^ ^,^ ^9^ ^,^ ^g^.

The objective of this research was to study a group of children or adolescents who had been victims of domestic violence assisted in specialized services, and a group of students without reports of domestic violence, to learn about the predictors of resilience, the quality and perception of social support, the resources present in the family environment and the chances of them developing mental health problems.

## METHODS

This is a cross-sectional, descriptive and analytical study, with 166 pairs formed by children and adolescents (9–16 years old) and their respective guardians. Of these children and adolescents, 100 were characterized as victims of domestic violence served in specialized services associated with the *Centro de Referência Especializado de Assistência Social* (Specialized Social Assistance Center of Reference – LICENSES) of the municipality of Campinas, state of São Paulo, constituting group 1 (G1). The remaining 66 were students of state schools located in the same regions as G1, without reports of domestic violence, constituting group 2 (G2). The children and adolescents of G1 suffered neglect of care or physical, psychological or sexual violence.

In both groups, intentional sampling was conducted^10^. The age group was chosen based on the instruments used and divided into four categories (9–10 years old, 11–12 years old, 13–14 years old and 15–16 years old); as second criterium, it was decided to divide the municipality into five regions (North, South, East, Southwest and Northwest), selecting an equal number of subjects per region; as last criterium, all kinds of domestic violence should have been present in each age group and region of G1. To select the subjects of G2, children or adolescents who attended schools in the same regions as G1 were sought for, so that it could be assumed that the subjects of both groups had the same level of exposure to situations of vulnerability.

As these were families in conditions of psychosocial vulnerability, many of which did not agree to participate in the research due to the theme, there was difficulty of adherence. Thus, at least 66 children or adolescents were chosen per group, 12 by region of the municipality, three by age group and one for each type of violence in G1.

Participants with previous diagnosis of neurological disease or intellectual disabilities were excluded.

### Instruments

To identify the subjects’ chances of developing mental health problems, the validated Brazilian version of the Strengths and Difficulties Questionnaire (SDQ) was used^11^. This is a widely used screening questionnaire, with positive psychometric indexes of validity and reliability in several countries, including Brazil. This instrument assesses the mental health problems of children and adolescents aged 4–16 years old, in three versions: one answered by children and adolescents aged 11–16 years old (self-application), one answered by the guardians and one answered by the teachers of subjects aged 4–16 years old. In addition, it assesses the capabilities and difficulties of these subjects, enabling their classification into three categories: normal (N), borderline (B) and abnormal (A) development. In this study, the self-application version, answered by the children and adolescents (SDQ-CA), as well as the one answered by their guardians (SDQ-G)^11^ were used.

To learn about the characteristics of resilience, the validated Brazilian version of the Resiliency Scales for Children and Adolescents (RSCA) of Prince-Embury^12^ was applied to the children and adolescents. This scale assesses individual attributes deemed as significant to the process of resilience in the 9–18 year-old age group, classifying it in: low, below average (BlA), average, above average (AbA) and high. It is composed of three scales: Scale I (SI – sense of control) assesses personal attributes such as optimism, self-efficacy and adaptability; Scale II (SII – relationship skills) assesses confidence, support, comfort and tolerance; and Scale III (SIII – emotional reactivity) evaluates sensitivity, recovery and damages. The psychometric parameters of the validated Brazilian version^12^ were satisfactory in relation to Prince-Embury's version in all three scales (Cronbach's alpha coefficients: SI – 0.83; SII – 0.90; SIII – 0.87), indicating good internal consistency of the instrument^12^.

To learn about the subjects’ support network, the validated Brazilian version of the Social Support Appraisals (SSA)^13^ was used. This instrument evaluates the perception of the social support received from family, friends, teachers and other people in the community of children and adolescents aged 9–18 years old, classifying it as low, medium and high. This is the first version of the SSA adapted to Brazilian Portuguese^13^, having showed appropriate psychometric parameters (alpha coefficient of the total scale: 0.79.)

A semi-structured questionnaire^10^ prepared by the researcher was applied to the guardians to characterize the population studied and exclude subjects who had experienced situations of domestic violence from G2.

The Home Environment Resources Scale (HERS)^14^, used with the parents, is a Brazilian instrument used to learn about the resources of the family environment: supervision and organization of routines, opportunities for interaction with the parents, presence of resources in the physical environment and characterization of the family. The HERS^14^ showed internal consistency, with appropriate psychometric parameters (alpha coefficient of the total scale: 0.74).

### Procedures

Data were collected from March 2014 to February 2016. The interviews were carried out simultaneously with the pairs (with the consent of the children or adolescents and their guardians) by two psychologists with experience in applying the instruments. In G1, 50% occurred on the families’ visitation day at the services; due to the high absenteeism, some were also carried out at their houses (26%) and at the school or socio-educational center frequented by the children and adolescents (24%). In G2, the interviews were carried out with the pairs at the schools that agreed to participate in the research.

This study was approved by the Research Ethics Committee of the School of Medical Sciences of Universidade Estadual de Campinas (Process 371,078), by the Municipal Secretariat of Citizenship and Social Inclusion (municipality of Campinas) and by the State Secretariat of Education (Regional Executive Boards of Education of Campinas).

### Data Analysis

The data were processed using the SPSS software version 16.0^15^, and presented in tables containing the absolute and relative frequencies.

To assess the association between the variables, the chi-square test or the Fisher-Freeman-Halton test were employed. In addition, the odds ratio (OR) was determined, the non-fitted OR having been calculated via univariate logistic regression (ENTER method). In this case, the dependent variable resilience, with its low, BlA, medium, AbA and high categories, was reduced to only two categories: low (low/BlA) and adequate (medium/ABa/high).

The fitted OR values were determined via multivariate logistic regression (Backward Stepwise “Wald” method), with p-value = 0.05 for inclusion and p-value = 0.10 for exclusion. All predicting variables with p-value < 0.20 were selected for the univariate analysis.

## RESULTS

Of the 166 children and adolescents in the study, 89 (53.6%) were female. All children and adolescents were studied, 100 in G1 and 66 in G2. In G1, 25 children of the total 100 belonged to each age group (9–10; 11–12; 13–14; 15–16 years), whereas in G2, from 15 to 18 of the total 66 belonged to each of them; as for region, in G1, 20 of the total 100 belonged to each region (North, South, East, Northwest and Southwest) and in G2, from 12 to 14 of the total 66 belonged to each of them.

In this study, 63% of the children or adolescents (G1) suffered more than one form of violence, psychological violence being the most frequent (82%), followed by neglect or abandonment (58%), sexual violence (26%) and physical violence (23%). The main aggressors were the parents, father and mother (50.9%) or only one of them (30%).

The distribution, with differences between the groups in relation to socioeconomic status and number of people per residence, is presented in Table 1.

**Table 1:** Socioeconomic conditions and number of inhabitants per household by group.

Socioeconomic conditions and family infrastructure		Group				Total		p
		G1 (n = 100)		G2 (n = 66)				
		n	%	n	%	n	%	
Guardian's labor situation								**0.012** a
	Formal job with labor rights	34	34.0	36	54.6	70	42.2	
	Steady job without labor rights	9	9.0	2	3.0	11	6.6	
	Regular self-employment	14	14.0	14	21.2	29	17.5	
	Irregular self-employment	10	10.0	3	4.5	12	7.2	
	Unemployed or retired	33	33.0	11	16.7	44	26.5	
Total household income (minimum wages)^c^								**< 0.001** a
	Less than 1	34	34.0	4	6.0	38	22.9	
	Between 1 and 2	40	40.0	13	19.7	53	31.9	
	Between 2 and 3	19	19.0	18	27.3	37	22.3	
	More than 3	7	7.0	31	47.0	38	22.9	
Social benefit								**0.002** a
	Yes	59	59.0	23	34.8	82	49.4	
	No	41	41.0	43	65.2	84	50.6	
Number of people living in the house								**0.113** a
	From 1 to 4	42	42.0	36	54.5	78	47.0	
	Five or more	58	58.0	30	45.5	88	53.0	
	Guardian's education level	98	100.0	63	100	161^d^	100	**< 0.001** b
	Illiterate	1	1.0	3	4.8	4	2.5	
	Elementary School I	35	35.7	11	17.5	46	28.6	
	Elementary School II	38	38.8	13	20.6	51	31.7	
	High School	20	20.4	30	47.6	50	31.1	
	Higher Education	4	4.1	6	9.5	10	6.2	

p: probability; G1: group 1; G2: group 2

a Probability estimated by the Chi-square test.

b Probability estimated by the Fisher-Freeman-Halton Test.

c Including social benefits.

d Five (5) guardians who did not provide this information were not included in the analysis.

Values with statistical significance are presented in bold.

As for the prevalence of resilience, G1 featured 72% of adequate and 28% of low resilience in SI, 92% of adequate and 8% of low resilience in SII, and 87% of adequate and 13% of low resilience in SIII. G2 featured 74.2% of adequate and 25.8% of low resilience in SI, 89.4% of adequate and 10.6% of low resilience in SII, and 98.5% of adequate and 1.5% of low resilience in SIII.

The prevalence of low resilience measured by the three resilience scales and distributed by group, socioeconomic conditions, number of people per residence, sex, age, SSA and RAF in the univariate analysis are presented in Tables 2, 3 and 4.

**Table 2:** Distribution of resilience evaluated by Scale I (sense of control) in relation to socioeconomic status, number of inhabitants per household, group, sex, age, SSA and HERS.

Variable		Low+BlA		Total	p	OR	95%CI
		n	%				
Group					0.750		
	G1	28	28.0	100		1.12	0.55–2.27
	G2	17	25.8	66		1.00	
Guardian's labor situation					0.911		
	Unemployed or retired	13	29.5	44		0.91	0.40–2.08
	Irregular self-employment	4	30.8	13		0.97	0.18–2.95
	Regular self-employment	6	21.4	28		0.60	0.21–1.67
	Steady job without labor rights	0	0	11		0.00	a
	Formal job with labor rights	22	31.4	70		1.00	
Income (minimum wages)					0.591		
	Less than 1	8	21.1	38		0.75	0.26–2.16
	Between 1 and 2	14	26.4	53		1.01	0.39–2.59
	Between 2 and 3	13	35.1	37		1.52	0.56–4.08
	More than 3	10	26.3	38		1.00	
Social benefit					0.261		
	No	26	31.0	84		1.49	0.74–2.97
	Yes	19	23.2	82		1.00	
Number of people living in the house					0.273		
	Five or more	27	30.7	88		1.48	0.74–2.96
	From 1 to 4	18	23.1	78		1.00	
Guardian's education level					0.876		
	Illiterate	1	25.0	4		1.33	0.09–20.71
	Elementary School I	12	26.1	46		1.41	0.26–7.60
	Elementary School II	12	23.5	51		1.23	0.23–6.60
	High School	16	32.0	50		1.88	0.36–9.90
	Higher Education	2	20.0	10		1.00	
Sex					0.693		
	Female	23	25.8	89		0.87	0.44–1.73
	Male	22	28.6	77		1.00	
Age (years)					0.937		
	9 to 10	12	30.0	40		1.11	0.43–2.86
	11 to 12	11	26.8	41		0.95	0.36–2.47
	13 to 14	10	23.8	42		0.81	0.30–2.14
	15 to 16	12	27.9	43		1.00	
SSA-Family					**0.019**		
	Low	12	42.9	28		**20.25**	**2.40–170.68**
	Mean	32	29.1	110		**11.08**	**1.44–85.01**
	High	1	3.6	28		1.00	
SSA-Friends					**0.001**		
	Low	11	55.0	20		**7.74**	**2.51–23.89**
	Mean	25	31.3	80		**2.88**	**1.23–6.72**
	High	9	13.6	66		1.00	
SSA-Teacher					**< 0.001**		
	Low	6	37.5	16		**5.47**	**1.57–19.07**
	Mean	31	44.9	69		**7.44**	**3.12–17.78**
	High	8	9.9	81		1.00	
SSA-Other					**< 0.001**		
	Low	12	57.1	21		**31.33**	**5.97–164.48**
	Mean	31	32.3	96		**11.21**	**2.56–49.15**
	High	2	4.1	49		1.00	
SSA-Total					**0.003**		
	Low	12	46.2	26		**41.14**	**4.91–344.52**
	Mean	32	35.2	91		**26.03**	**3.43–197.53**
	High	1	2.0	49		1.00	
HERS-Routine		–	–	–	0.603	1.01	0.99–1.03
HERS-Interaction		–	–	–	0.931	1.00	0.98–1.02
HERS-Resources		–	–	–	0.755	1.00	0.98–1.02
HERS-Total		–	–	–	0.745	1.00	0.98–1.03

BlA: below average; p: probability estimated by the Wald test; G1: Group 1; G2: Group 2; SSA: Social Support Appraisals; HERS: Home Environment Resources Scale

a Undetermined.

Values with statistical significance are presented in bold.

**Table 3:** Distribution of resilience evaluated by Scale II (relationship skills) in relation to socioeconomic status, number of inhabitants per household, group, sex, age, SSA and HERS.

Variable		Low+BlA		Total	p	OR	95%CI
		n	%				
Group					0.568		
	G1	8	8.0	100		0.73	0.25–2.13
	G2	7	10.6	66		1.00	
Guardian's labor situation							
	Unemployed or retired	3	6.8	44		0.57	0.14–2.26
	Irregular self-employment	0	0	12		0.00	a
	Regular self-employment	4	14.3	28	0.896	1.29	0.36–4.69
	Steady job without labor rights	0	0	11		0.00	a
	Formal job with labor rights	8	11.4	70		1.00	
Income (minimum wages)							
	Less than 1	2	5.3	38		0.37	0.07–2.02
	Between 1 and 2	4	7.5	53	0.646	0.54	0.13–2.16
	Between 2 and 3	4	10.8	37		0.80	0.20–3.25
	More than 3	5	13.2	38		1.00	
Social benefit							
	No	7	8.3	84	0.749	0.84	0.29–2.44
	Yes	8	9.8	82		1.00	
Number of people living in the house							
	Five or more	11	12.5	88	0.109	2.64	0.81–8.67
	From 1 to 4	4	5.1	78		1.00	
Guardian's education level							
	Illiterate	0	0	4		0.00	a
	Elementary School I	5	10.9	46		1.10	0.11–10.57
	Elementary School II	4	7.8	51	0.991	0.77	0.08–7.67
	High School	5	10.0	50		1.00	0.10–9.61
	Higher Education	1	10.0	10		1.00	
Sex					0.573		
	Female	7	7.9	89		0.74	0.25–2.13
	Male	8	10.4	77		1.00	
Age (years)					0.139		
	9 to 10	1	2.5	40		0.19	0.02–1.75
	11 to 12	7	17.1	41		1.56	0.45–5.39
	13 to 14	2	4.8	42		0.38	0.07–2.08
	15 to 16	5	11.6	43		1.00	
SSA-Family					0.153		
	Low	6	21.4	28		a	a
	Mean	9	8.2	110		a	a
	High	0	0	28		1.00	
SSA-Friends					**< 0.001**		
	Low	9	45.0	20		**12.68**	**3.32–48.48**
	Mean	2	2.5	80		**0.40**	**0.07–2.24**
	High	4	6.1	66		1.00	
SSA-Teacher					0.001		
	Low	6	37.5	16		15.60	3.36–72.36
	Mean	6	8.7	69		2.48	0.60–10.30
	High	3	3.7	81		1.00	
SSA-Other					0.001		
	Low	7	33.3	21		24.00	2.72–211.93
	Mean	7	7.3	96		3.78	0.45–31.59
	High	1	2.0	49		1.00	
SSA-Total					**< 0.001**		
	Low	9	34.6	26		25.41	2.99–215.72
	Mean	5	5.5	91		2.79	0.32–24.59
	High	1	2.0	49		1.00	
HERS-Routine		–	–	–	0.805	1.00	0.97–1.04
HERS-Interaction		–	–	–	0.162	0.98	0.95–1.01
HERS-Resources		–	–	–	0.401	1.01	0.98–1.05
HERS-Total		–	–	–	0.965	1.00	0.96–1.01

BlA: below average; p: probability estimated by the Wald test; G1: Group 1; G2: Group 2; SSA: Social Support Appraisals; HERS: Home Environment Resources Scale

a Undetermined.

Values with statistical significance are presented in bold.

**Table 4:** Distribution of resilience evaluated by Scale III (emotional reactivity) in relation to socioeconomic status, number of inhabitants per household, group, sex, age, SSA and HERS.

Variable		Low+BlA		Total	p	OR	95%CI
		n	%				
Group					**0.030**		
	G1	13	13.0	100		**9.71**	**1.24–76.14**
	G2	1	1.5	66		1.00	
Guardian's labor situation							
	Unemployed or retired	5	11.4	44		2.12	0.54–8.35
	Irregular self-employment	2	15.4	13	0.746	3.00	0.48–18.39
	Regular self-employment	2	7.1	28		1.27	0.22–7.36
	Steady job without labor rights	1	9.1	11		1.65	0.17–16.29
	Formal job with labor rights	4	5.7	70		1.00	
Income (minimum wages)							
	Less than 1	3	7.9	38		1.54	0.24–9.80
	Between 1 and 2	8	15.1	53	0.216	3.20	0.64–16.01
	Between 2 and 3	1	2.7	37		0.50	0.04–5.76
	More than 3	2	5.3	38		1.00	
Social benefit							
	No	5	6.0	84	0.251	0.51	0.16–1.60
	Yes	9	11.0	82		1.00	
Number of people living in the house							
	Five or more	7	8.0	88	0.814	0.88	0.29–2.62
	From 1 to 4	7	9.0	78		1.00	
Guardian's education level							
	Illiterate	1	25.0	4		3.00	0.14–64.26
	Elementary School I	2	4.3	46		0.41	0.03–5.01
	Elementary School II	6	11.8	51	0.511	1.20	0.13–11.21
	High School	3	6.0	50		0.57	0.05–6.16
	Higher Education	1	10.0	10		1.00	
Sex					**0.015**		
	Female	13	14.6	89		**13.00**	**1.66–101.86**
	Male	1	1.3	77		1.00	
Age (years)					0.131		
	9 to 10	3	7.5	40		0.31	0.08–1.23
	11 to 12	0	0.0	41		a	a
	13 to 14	2	4.8	42		0.19	0.04–0.93
	15 to 16	9	20.9	43		1.00	
SSA-Family					**0.001**		
	Low	8	28.6	28		**10.80**	**1.25–93.44**
	Mean	5	4.5	110		1.29	0.14–11.47
	High	1	3.6	28		1.00	
SSA-Friends					0.676		
	Low	2	10.0	20		1.72	0.29–10.18
	Mean	8	10.0	80		1.72	0.49–6.00
	High	4	6.1	66		1.00	
SSA-Teacher					**0.008**		
	Low	5	31.3	16		**8.75**	**2.03–37.63**
	Mean	5	7.2	69		1.50	0.39–5.84
	High	4	4.9	81		1.00	
SSA-Other					0.148		
	Low	4	19.0	21		5.53	0.93–32.98
	Mean	8	8.3	96		2.14	0.44–10.47
	High	2	4.1	49		1.00	
SSA-Total					**0.024**		
	Low	6	23.1	26		**7.05**	**1.31–37.97**
	Mean	6	6.6	91		1.66	0.32–8.55
	High	2	4.1	49		1.00	
HERS-Routine		–	–	–	**< 0.001**	**0.94**	**0.90–0.97**
HERS-Interaction		–	–	–	**0.002**	**0.95**	**0.92–0.98**
HERS-Resources		–	–	–	0.321	0.98	0.95–1.02
HERS-Total		–	–	–	**0.001**	**0.93**	**0.89–0.97**

BlA: below average; p: probability estimated by the Wald test; G1: Group 1; G2: Group 2; SSA: Social Support Appraisals; HERS: Home Environment Resources Scale

a Undetermined.

Values with statistical significance are presented in bold.

As in the analysis of resilience no other differences between G1 and G2 were found, it was decided to group them for studying the factors that could interfere in the construction of this skill.

After the multivariate analysis, only variables SSA-teacher and SSA-other remained in SI; only SSA-other remained in SII; and only sex, SSA-teacher and HERS-routine remained in SIII. In all of them, a low perception of social support was associated with a higher chance of low resilience. As for emotional reactivity (SIII), girls were 15.49 times more likely to show low resilience than boys (Table 5).

**Table 5:** Final multivariate model of low resilience (low/BlA) in the three Scales for sex, SSA and HERS.

Resiliency questionnaire	Variable independent	p	Adjusted OR	95%CI
	SSA-Teacher	**0.010**		
Scale I	Low		2.84	0.74–10.85
(sense of control)	Mean		**4.19**	**1.66–10.57**
	High		1.00	
	SSA-Other	**0.012**		
Scale I	Low		**14.08**	**2.47–80.36**
(sense of control)	Mean		**6.81**	**1.49–31.18**
	High		1.00	
	SSA-Other	**0.033**		
Scale II	Low		**17.45**	**1.26–242.29**
(relationship	Mean		1.00	
skills)	High		1.00	
	Sex	**0.021**		
Scale III	Female		**15.49**	**1.50–160.10**
(emotional reactivity)	Male		1.00	
	SSA-Teacher	**0.019**		
Scale III	Low		**37.16**	**2.89–478.43**
(emotional reactivity)	Mean		1.88	0.36–9.69
	High		1.00	
Scale III (emotional reactivity)	HERS-Routine	**0.005**	**0.94**	0.90–0.98

BlA: below average; p: probability estimated by the Wald test; SSA: Social Support Appraisals; HERS: Home Environment Resources Scale.

Values with statistical significance are presented in bold.

SDQ-C/A was applied to 126 subjects (75 from G1 and 51 from G2), while SDQ-G was applied to all 166. The SDQ-C/A analysis showed that, in G1, 52% (39/75) could be placed in the abnormal (27/75) or borderline (12/75) categories, while in G2, this occurred in 33.3% (17/51) of the subjects (abnormal: 11; borderline: 6) (p = 0.112).

As for SDQ-R, 64% (64/100) of G1 could be placed in categories A (41/100) and L (23/100), while in G2, this occurred in 37.9% (25/66) of the subjects (abnormal: 20/66; borderline: 5/66) (p = 0.002).

In addition, the subjects of G1 and G2 with low resilience in SI of the RSCA were 4.9 times more likely to develop mental health problems (A) in SDQ-C/A [OR = 4.90 (95%CI 1.66–14.44) (p = 0.008)]. In the other scales, no association between low resilience and mental health problems was observed.

## DISCUSSION

Despite advances in the research on resilience, they are recent, with controversies and disagreements as to the definitions, concepts and research methods, which complicates the comparison of data^14^. Current research with populations of children and adolescents in situations of social vulnerability carried out in Latin American countries showed prevalence rates corresponding to 71.7% and 65.8% resilience, using different instruments^16^ ^,^ ^17^.

In the results of this study, the children and adolescents who had been victims of violence (G1), although exposed to traumatic situations, showed a prevalence of adequate resilience corresponding to 72% (SI), 92% (SII) and 87% (SIII), and of low resilience corresponding to 28% (SI), 8% (SII) and 13% (SIII) (Tables 2, 3 and 4). These data are similar to those of other studies with subjects in situations of social vulnerability^16^ ^,^ ^17^ and also to those of G2, with a small difference in SIII (1.5% in the low category) (Table 4).

Another research^18^ performed with a group of institutionalized children exposed to interparental violence, compared to a group of subjects without violations of rights, found levels of resiliency, coping and social competence that were considered adequate (using other instruments). There were no differences between the groups, except for higher frequency of coping strategies in the first group and for these having been perceived by the children as more effective. Acting out-type strategies (situations of physical and verbal aggression) were also more frequent in the first group and the children recognize them as more effective, compared to unexposed children, despite them being ineffective strategies.

In the multivariate analysis (Table 5), low perception of social support (other people in the community and teachers) was also associated with a greater chance of low resilience.

Subjects with low perception of support by other people in the community had more chances of low resilience in the personal attributes of optimism, adaptability and self-efficacy (SI) and in relationship skills (SII) (Table 5). Another study examined the influence of people considered significant in the community on the construction of aspects that predict resilience in children and adolescents who have been victims of domestic violence; according to this study, these figures play a moderating role in the subjects’ cognitive ability and home stability^19^. The functions performed by role models (even more distant ones, like people in the community) and by the social support network are fundamental protective factors in the construction of resilience^20^.

The school and the teachers were identified as major sources of protection for the subjects of this study (Table 5). It was found that children and adolescents with smaller perception of support from the teacher had higher chances of low resilience in the personal attributes of optimism, self-efficacy and adaptability (SI) and in emotional reactivity (SIII). The perception of support from the teacher is crucial, and the school is as important source of support, generating feelings of greater safety, especially for children/adolescents in situation of vulnerability and with weakened role models in their families. Thus, distancing in teacher-student relationships may be considered a risk factor^21^.

The literature indicates the family as the main source of social support^20^, and family stability and social relations as important predictors of a more adaptive and resilient emotional competence^22^. Surprisingly, as opposed to these researches, the multivariate analysis of this study did not identify it as predictive of resilience, neither as a vulnerability factor in any of the RSCA scales^12^. The hypothesis is that the conjunction of so many concurrent problems in these families hinders the perception of this support by the subjects of the study, which does not mean to say the role of support they can exercise should be ruled out.

It was found that families in situation of domestic violence (G1) had higher socioeconomic vulnerability compared to those in G2, but these aspects are not associated with the predictors of resilience. Precarious socioeconomic conditions can aggravate the situations of violence and also the risk of problems in the psycho-emotional development of children and adolescents^23^ ^,^ ^24^.

It was found through the RAF^14^ that children and adolescents who had rules or routine in their lives showed lower chances of low resilience in emotional reactivity (SIII) (Table 5). Parents with negligent characteristics cannot establish a routine for their children, which hampers the construction of resilience in general. Therefore, the function of social support through the establishment of rules or routines for the children, in which the family has a fundamental role, generates feelings of security and helps the subjects process their emotions, avoiding the onset of mental disorders^9^ ^,^ ^12^ ^,^ ^20^.

The girls of this study had higher chances of low resilience in emotional reactivity (SIII). This index shows the ability to manage or regulate emotions, to maintain one's balance in stressful situations^12^. It is assumed, from the discussions about gender issues, that girls of both groups may be experiencing situations of higher risk, which increase their emotional limitations or make them react in self-defense. Having been victim of sexual abuse in childhood and being raised by guardians with anti-social behavior or some kind of dependency on psychoactive substances are indicated as risk factors for conduct disorders among girls^18^ ^,^ ^25^.

The literature shows that the prevalence of mental disorders in children and adolescents varies between 10% and 20%^26^.

The prevalence of chances of mental health problems found in G1 using both versions of the SDQ were quite high (52% in SDQ-C/A and 64% in SDQ-G). In another research^3^, these authors found similar data using the SDQ, with the prevalence of chances of mental health problems in children and adolescents who had been victims of domestic violence having corresponded to 65.6%, compared to the general population.

However, the rates presented by G2 were also high (33.3% in SDQ-C and 37.9% in SDQ-G), compared to the average of the population. These rates can be associated with the fact that the children and adolescents of G2 had also been exposed to possible situations of social or community violence, poor health and housing conditions, economic and cultural difficulties, low educational level of the guardians, among other situations of vulnerability. These factors may affect the subjects’ global and emotional development, causing these high rates of emotional symptoms^1^ ^,^ ^20^.

A research^27^ that used the Child Behavior Checklist observed a sharp increase in internalizing and externalizing mental health problems, especially in children whose mothers have low education level and in families with greater socioeconomic vulnerability, corroborating studies conducted in other countries.

Domestic violence against children and adolescents was shown to be a risk factor for greater chance of mental health problems in the subjects studied, based on the evaluation of SDQ-G (p = 0.002), including in G2. This result is relevant for studies in the field of children's mental health^2^ ^,^ ^3^ ^,^ ^28^.

In addition, it was found that the children and adolescents from both groups with low resilience in SI (sense of control), which assesses individual strategies to deal with stressful events (optimism, self-efficacy and adaptability^12^) were 4.9 times more likely to develop mental health problems in SDQ-C/A. This indicates an association between less resilient subjects and higher prevalence of emotional symptoms. The hypothesis is that children and adolescents, regardless of the group in which they were inserted, experience the consequences of a frail external social support, as it assists in the construction of resilience and in the protection against the early onset of emotional disorders. Social support, allied to healthy educational practices, helps moderating the negative effects of situations of vulnerability and protects the children against precarious care, helplessness, violence and isolation^20^ ^,^ ^21^.

The high absenteeism in the interviews in both groups, due to the social vulnerability of the population as a whole and to the theme chosen, was a limitation of the study. However, the importance of carrying out studies in the field of domestic and social or community violence should be emphasized, despite the difficulties of access to this population, for the construction of more effective public service policies.

The resilience rates shown by the subjects of this study (Tables 2, 3 and 4) indicate good perspectives for the future, though they are neither uniform nor static – an individual can be resilient in some respects and not in others, or even show resiliency in different ways in the various stages of life^22^. At the same time, they expressed suffering through important emotional symptoms, those subjected to domestic violence having shown more serious problems. Specialized services may be exerting a role in the construction of predictors of resilience for the children and adolescents in G1; however, because they experienced traumatic situations, they showed important emotional problems^1^ ^-^ ^3^ ^,^ ^20^. In relation to G2, the chance of mental health problems may be associated also with the possible exposure to social and community violence^7^.

Therefore, reflecting about the aspects that influence the construction of resilience and situations of violence may contribute so that mental disorders are avoided or do not become chronic^29^. In addition, the importance of investing in early intervention strategies^7^ ^,^ ^29^ aimed at promoting the quality of the relationship between children and adults is emphasized. The presence of role models with adequate conditions for offering social support can assist in the protection of their mental health and strengthen their resilience, which is dynamic and can change according to the intrinsic characteristics of the subject or the social relationships experienced.

## CONCLUSIONS

Violence may have increased the chance of development of mental health problems, domestic violence being an aggravating factor. In this study, it was identified that girls had higher chances of low resilience in emotional reactivity, i.e., difficulties to cope and recover emotionally from a situation of vulnerability. Children and adolescents with low resilience in relation to emotional reactivity were more likely to develop mental health problems.

The absence of perception of social support from the teacher and other people in the community of the children and adolescents was also identified as vulnerability or risk factor, being associated with low resilience. On the other hand, the presence of routine or rules established by the guardians was considered a protective factor.

The perception of adequate social or emotional support can be an important protective factor and facilitate the development of resiliency. Investments in psychosocial work and the strengthening of effective actions based on the concept of a care network for families in situation of violence can be strategies for the development of predictors of resilience.

There is need for research on aspects that predict resilience and investment in intervention strategies for this population, as a way to promote mental health.

## Footnotes

a Organização Mundial da Saúde. Relatório mundial sobre a prevenção da violência 2014. São Paulo: Núcleo de Estudos da Violência da Universidade de São Paulo; 2014 [cited 2018 Mar 8]. Available from: apps.who.int/iris/bitstream/10665/145086/5/9789241564793_por.pdf

b Waiselfisz JJ. Mapa da violência 2012: crianças e adolescentes do Brasil. Rio de Janeiro: FLACSO Brasil; 2012 [cited 2018 Mar 8]. Available from: https://www.mapadaviolencia.org.br/pdf2012/MapaViolencia2012_Criancas_e_Adolescentes.pdf

c Azevedo MA, Guerra VNA. Violência doméstica contra crianças e adolescentes: um cenário em desconstrução. In: UNICEF Brasil. Direitos negados. Brasília (DF); 2006 [cited 2018 Mar 8]. Available from: https://www.unicef.org/brazil/pt/Cap_01.pdf

d Munist M, Santos LH, Kotliarenco MA, Suárez Ojeda EN, Infante F, Grotberg E. Manual de identificación y promocion de la resiliencia en niños y adolescentes. Washington (DC): Organización Panamericana de la Salud; Organización Mundial de La Salud; Fundación W.K. Kellogg; Autoridad Sueca para el Desarrollo Internacional; 1998 [cited 2018 Mar 8]. Available from: http://www1.paho.org/hq/dmdocuments/2009/Resilman.pdf

e Ministério da Saúde (BR), Secretaria de Atenção à Saúde, Departamento de Ações Programáticas e Estratégicas. Linha de cuidado para a atenção integral à saúde de crianças, adolescentes e suas famílias em situação de violência: orientação para gestores e profissionais de saúde. Brasília (DF); 2012 [cited 2018 Mar 8]. (Série F. Comunicaçãao e Educação em Saúde). Available from: http://bvsms.saude.gov.br/bvs/publicacoes/linha_cuidado_criancas_familias_violencias.pdf

f Munist M, Santos LH, Kotliarenco MA, Suárez Ojeda EN, Infante F, Grotberg E. Manual de identificación y promocion de la resiliencia en niños y adolescentes. Washington (DC): Organización Panamericana de la Salud; Organización Mundial de La Salud; Fundación W.K. Kellogg; Autoridad Sueca para el Desarrollo Internacional; 1998 [cited 2018 Mar 8]. Available from: http://www1.paho.org/hq/dmdocuments/2009/Resilman.pdf

g Ministério da Saúde (BR), Secretaria de Atenção à Saúde, Departamento de Ações Programáticas e Estratégicas. Linha de cuidado para a atenção integral à saúde de crianças, adolescentes e suas famílias em situação de violência: orientação para gestores e profissionais de saúde. Brasília (DF); 2012 [cited 2018 Mar 8]. (Série F. Comunicação e Educação em Saúde). Available from: http://bvsms.saude.gov.br/bvs/publicacoes/linha_cuidado_criancas_familias_violencias.pdf
